# Alpha-Tocopherol Protects Porcine Oocytes from Acetamiprid-Induced Meiotic Defects by Alleviating Oxidative Stress-Mediated Ferroptosis

**DOI:** 10.3390/antiox14111304

**Published:** 2025-10-30

**Authors:** Yanhong Liu, Yijing He, Miaoyu Chen, Qinfeng Sun, Biao Zhang, Genkui Zhang, Aiqiao Cao, Qiao Li, Weihan Wang, Shiqiang Ju

**Affiliations:** 1 MOE Joint International Research Laboratory of Animal Health and Food Safety, College of Veterinary Medicine, Nanjing Agricultural University, Nanjing 210095, China; 2022807170@stu.njau.edu.cn (Y.L.); 2019107093@njau.edu.cn (Y.H.); 2023807146@stu.njau.edu.cn (M.C.); 2023107095@stu.njau.edu.cn (Q.S.); 2023107104@stu.njau.edu.cn (B.Z.); 2023807210@stu.njau.edu.cn (G.Z.); liqiao@njau.edu.cn (Q.L.); 2 Shenzhen Agricultural Product Quality Safety Inspection and Testing Center, Shenzhen 518000, China; aiqiao@163.com

**Keywords:** porcine oocyte, acetamiprid, alpha-tocopherol, lipid peroxidation, ferroptosis

## Abstract

Acetamiprid (ACE), a widely used neonicotinoid insecticide, has raised concerns due to its potential reproductive toxicity. While its adverse effects on animal reproductive systems have been documented, the impact of ACE on mammalian oocytes remains poorly understood. This study aimed to investigate the potential effects of ACE exposure on porcine oocytes and evaluate whether alpha-tocopherol (α-TOC), a fat-soluble antioxidant, could alleviate ACE-induced oocyte damage. Porcine cumulus oocyte complexes (COCs) were exposed to ACE alone or co-treated with α-TOC for 44 h during in vitro maturation. ACE exposure significantly reduced the first polar body (PB1) excretion rate, arrested meiotic progression, and disrupted spindle assembly in porcine oocytes. Furthermore, ACE impaired mitochondrial function, evidenced by decreased mitochondrial membrane potential (MMP), while increasing intracellular reactive oxygen species (ROS) accumulation and lipid peroxidation (LPO). Additionally, ACE exposure induced intracellular iron overload and dysregulated ferroptosis-related genes, downregulating solute carrier family 7 member 11 (SLC7a11) and glutathione peroxidase 4 (GPX4) while upregulating transferrin receptor 1 (TfRC) and acyl-CoA synthetase long-chain family member 4 (ACSL4), contributing to the occurrence of oocyte ferroptosis. Notably, α-TOC co-treatment effectively alleviate oxidative stress and lipid peroxidation, thereby protecting oocytes from ACE-induced ferroptosis. Collectively, these findings indicate that oxidative stress-mediated ferroptosis may be a major contributing pathway through which ACE impairs oocyte maturation and suggest that α-tocopherol may serve as a protective agent against ACE-induced oocyte damage.

## 1. Introduction

The widespread application of insecticides has greatly enhanced agricultural efficiency and facilitated a more productive lifestyle. However, the environmental contamination stemming from insecticide residues poses a threat to both human and animal health. The development of insect resistance to conventional organophosphorus pesticides has promoted the development of neonicotinoid insecticides [1]. In recent years, acetamiprid (ACE), as a new generation of neonicotinoid insecticides [2] and a primary alternative to organophosphorus insecticides, has been widely used in agricultural production [3]. While the widespread use of ACE has brought significant production benefits to modern agriculture, its potential toxic effects on mammals, especially health threats to their reproductive systems, have received increasing attention. ACE is stable in structure, soluble in water, and can persist in water, soil, and crops for a long time without degradation [4]. Consequently, animals can accumulate ACE in their bodies by consuming ACE-contaminated agricultural products or coming into contact with contaminated soil and water, inducing toxic effects and potentially passing it to their offspring through the placental barrier [5]. ACE exerts its insecticidal activity against insects by acting as an acetylcholine receptor agonist; it has been shown to bind mammalian acetylcholine receptors with significant affinity [6]. Importantly, mammalian nicotinic acetylcholine receptors are expressed in the reproductive system [7,8], which has raised great concerns about the reproductive toxicity of ACE in mammals. Previous studies have established that ACE exposure is associated with reproductive deficits in mammals [9]. ACE exposure leads to decreased testosterone levels, testicular tissue damage, and decreased sperm motility in male rats [10,11]. It also causes placental and fetal weight loss in pregnant female rats [12]. ACE exposure can cause histological damage to the mouse ovary, increase the number of atretic follicles, and impair early embryonic development [13,14]. Additionally, the cytotoxic effects of ACE are primarily attributed to oxidative stress and lipid peroxidation [15,16].

Alpha-tocopherol (α-TOC), the most biologically active form of vitamin E, is a lipid-soluble antioxidant with high bioavailability and antioxidant activity [17]. As it cannot be synthesized endogenously, α-TOC must be obtained through dietary intake [18]. Functionally, α-TOC exerts its antioxidant effects by intercepting peroxyl radicals, which are formed instantaneously when a lipid radical reacts with oxygen [19]. α-TOC plays a crucial role in the female reproductive system, and its deficiency can lead to infertility [20]. Studies have reported that α-TOC supplementation alleviates oxidative stress in rat testis and ovaries, reduces the number of atresia follicles, improves the morphology and function of rat ovaries, and increases sperm activity and survival rates [21,22]. In vitro, α-TOC protects the embryos from oxidative damage and promotes development in mouse and bovine systems [23,24].

Though multiple aspects of the reproductive toxicity of ACE exposure on the animal reproductive system have been revealed, there remains a paucity of evidence on the adverse effects of ACE on mammalian oocytes. To date, studies on the female reproductive toxicity of ACE have been primarily confined to rodent models [13]. In this study, porcine oocytes were used as a model to directly address the potential effects of ACE exposure on oocyte meiotic maturation. Additionally, the potential protective effects of α-TOC on ACE-exposed oocyte in vitro maturation were also explored.

## 2. Materials and Methods

### 2.1. Antibodies and Chemicals

The FACL4 rabbit monoclonal antibody (CY10198), SLC7a11 rabbit monoclonal antibody (CY7046), GPX4 rabbit monoclonal antibody (CY6959), and Transferrin Receptor 1 antibody (CY6618) were purchased from Abways (Shanghai, China). The beta Actin antibody was purchased from Servicebio (Wuhan, China). Acetamiprid (ACE, HY-B0823, purity 99.88%) and α-Tocopherol (HY-N0683, purity 98.88%) were purchased from MedChemExpress (Monmouth Junction, NJ, USA). Ferrostatin-1 (Fer-1, S7243, purity 99.98%) was purchased from Selleck (Houston, TX, USA). Hoechst 33342 and anti-α-tubulin-FITC antibody were purchased from Sigma-Aldrich (St. Louis, MO, USA). The HRP-labeled goat anti-rabbit IgG (A0208) and mitochondrial membrane potential assay kit (JC-1, C2006) were purchased from Beyotime Biotechnology (Shanghai, China). FerroOrange (F374) and Liperfluo (L248) were purchased from Dojindo Molecular Technologies Inc. (Shanghai, China).

### 2.2. Oocyte Collection and In Vitro Maturation (IVM)

Porcine ovaries were collected from a local abattoir (Nanjing, China) and transported to the laboratory in sterile 0.9% (*w*/*v*) physiological saline within 2 h. The cumulus oocyte complexes (COCs) were extracted from the follicles with diameters of 3 to 6 mm. Oocytes exhibiting homogeneous cytoplasm and surrounded by multiple layers of compact cumulus cells were selected for culture. The COCs were cultured in TCM-199 medium supplemented with 10 IU/mL PMSG and hCG, 10 ng/mL EGF, 0.57 mM L-cysteine, 0.91 mM sodium pyruvate, 3.05 mM D-glucose, 26.19 mM NaHCO_3_, 0.1% (*w*/*v*) polyvinyl alcohol, 7.5 mg/mL penicillin, 10% (*v*/*v*) porcine follicular fluid (PFF), and 5.0 mg/mL streptomycin. After 44 h of culture at 38.5 °C, the cumulus cells surrounding the oocytes were removed with 0.1% hyaluronidase, and the denuded oocytes were used for further experiments.

### 2.3. ACE Exposure and α-TOC Co-Treatment

The ACE, Fer-1, and α-TOC powders were each dissolved in dimethyl sulfoxide (DMSO) to prepare stock solutions of 449.1 mM, 50 mM, and 232.17 mM, respectively, and stored at −80 °C according to the manufacturer’s instructions. The ACE stock solution was diluted to working concentrations of 10, 20, and 40 μM with TCM-199 IVM medium for ACE exposure during in vitro culture of oocytes. For the co-treatment, Fer-1 or α-TOC stock solutions were diluted in IVM medium containing 20 μM ACE to achieve final concentrations of 2.5, 5, and 10 μM (Fer-1) or 5, 10, and 20 μM (α-TOC), respectively. The final concentration of DMSO in the culture system was less than 0.1%, which is within a safe range [25,26]. The selection of ACE concentrations was based on previous studies on *Xenopus laevis* embryos and human trophoblasts [27,28]. Additionally, we also referenced existing literature concerning ACE concentrations in acute blood [29,30]. The α-TOC concentrations were chosen based on previous studies on bovine oocyte maturation and embryo development [31]. The control group received an equivalent volume of DMSO.

### 2.4. Immunofluorescence Staining

The oocytes were fixed with 4% (*w*/*v*) paraformaldehyde for 30 min at room temperature (RT), and permeabilized with 0.1% (*v*/*v*) Triton X-100 for 8 h at RT. After being blocked with BSA 1% (*w*/*v*) at 37 °C for 2 h, the oocytes were incubated with α-tubulin-FITC antibody dilution (1:200) for 2 h, followed by incubation with Hoechst 33342 (1:100) for 15 min at 37 °C. Finally, the oocytes were mounted on glass slides with glycerol and examined using a laser confocal scanning microscope (Zeiss LSM 700 META, Oberkochen, Germany).

### 2.5. Assessment of Reactive Oxygen Species (ROS)

The ROS levels in oocytes were analyzed using a Reactive Oxygen Species Assay Kit (S0033, Beyotime Biotechnology, Shanghai, China). Briefly, oocytes were incubated in 10 μM DCFH-DA (Dichlorofluorescein diacetate) working solution at 37 °C and 5% CO_2_ for 30 min. After three washes with PBS, the fluorescence signals of oocytes were immediately analyzed via confocal fluorescence microscopy.

### 2.6. Mitochondrial Membrane Potential (MMP) Detection

The MMP in oocytes was assessed using a JC-1 mitochondrial membrane potential kit. The oocytes were transferred to pre-equilibrated JC-1 working solution and incubated at 37 °C with 5% CO_2_ for 30 min, following the kit’s instructions. Subsequently, the fluorescent signals of the oocytes were examined using a laser confocal scanning microscope.

### 2.7. Iron Assay

The Fe^2+^ levels in oocytes were analyzed using a fluorescent probe, FerroOrange. The oocytes were transferred to a 5 μM FerroOrange fluorescent probe working solution and incubated at 37 °C and 5% CO_2_ for 30 min. Subsequently, the fluorescent signals were visualized using a laser confocal scanning microscope.

### 2.8. Malondialdehyde (MDA) Assay

The MDA content in oocytes was determined using an MDA assay kit (S0131, Beyotime Biotechnology, Shanghai, China), following the manufacturer’s instructions. A total of 350 oocytes from each group were collected and lysed in RIPA lysis buffer containing 1 mM phenylmethylsulfonyl (abs812852, Absin, Shanghai, China) on ice for 30 min. The lysates were mixed with thiobarbituric acid (TBA) reagent and incubated at 100 °C for 15 min, followed by rapid cooling on ice. The absorbance of each sample was measured at 532 nm using a microplate reader.

### 2.9. Lipid Peroxidation (LPO) Imaging

The LPO levels in oocytes were analyzed using a liperfluo lipid peroxide fluorescent probe (L248, Dojindo Molecular Technologies Inc., Shanghai, China). The oocytes were incubated in 25 μM Liperfluo working solution at 37 °C with 5% CO_2_ for 30 min. After incubation, the fluorescence signals of LPO were detected and visualized using a laser confocal scanning microscope.

### 2.10. Quantitative Real-Time PCR (qRT-PCR)

Total RNA of 100 oocytes in each group was extracted using a SteadyPure Universal RNA Extraction Kit (AG21017, Accurate Biology, Changsha, China) and then synthesized into cDNA. qRT-PCR was conducted using reverse transcribed cDNA as a template on a Real-time PCR instrument (QuantStudio 6 Flex, Thermo Fisher Scientific (Waltham, MA, USA)). Primer sequences are listed in Supplementary Table S1. The data were analyzed using the 2^−ΔΔCt^ method.

### 2.11. Western Blot Analysis

A total of 150 porcine oocytes in each group were transferred into the RIPA lysates and boiled at 100 °C for 10 min. The lysates were stored at −80 °C until use. Protein samples were separated using protein prefabricated glue (ET15012Gel, ACE Biotechnology, Changzhou, China) and transferred to a polyvinylidene fluoride (PVDF) membrane. The membranes were blocked with no protein fast blocking solution (G2052, Servicebio, Wuhan, China) and then incubated with primary antibodies at 4 °C for 8 h and HRP-labeled secondary antibodies at 37 °C for 1 h. Finally, protein bands were visualized with an enhanced chemiluminescence (ECL) substrate (BL520A, Bioshap Technology Co., Ltd., Hefei, China).

### 2.12. Statistical Analysis

All experiments were repeated at least 3 times. One-way ANOVA was used to evaluate the differences between groups using SPSS 22.0, along with Duncan’s multiple comparisons tests. The results are presented as mean ± standard error (SE) values. Differences of *p* < 0.05 were considered significant.

## 3. Results

### 3.1. ACE Exposure Results in Defects in the Meiotic Maturation of Porcine Oocytes

**Figure 1:**
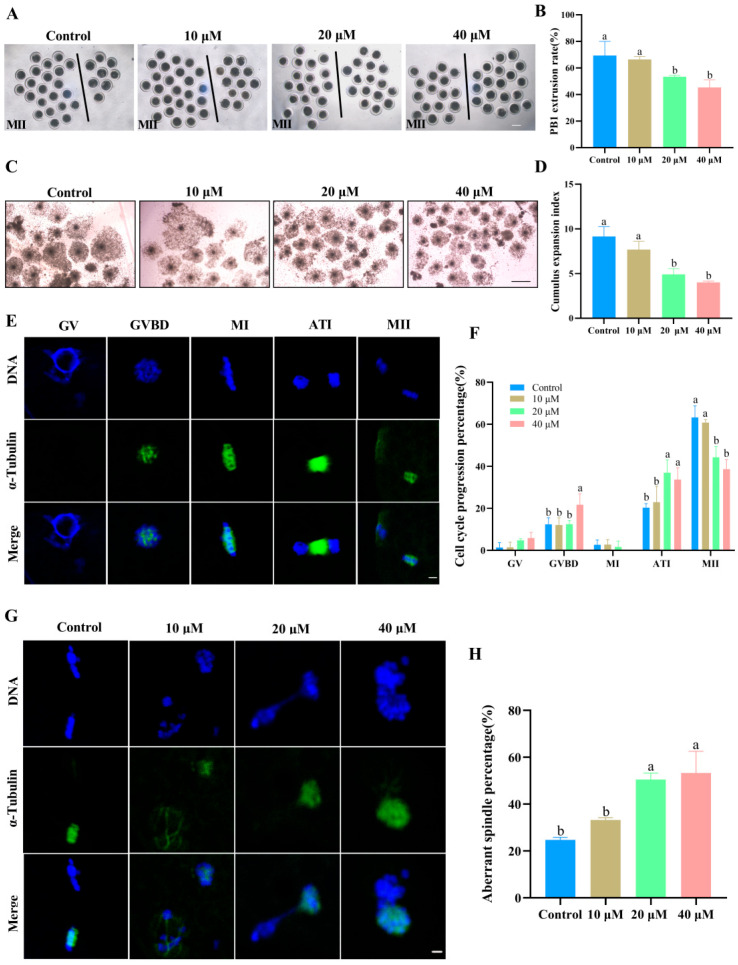
(**A**) The first polar body (PB1) expulsion of oocytes from the control group and ACE-treated groups (10, 20, and 40 μM). Oocytes to the left of the black line have successfully extruded PB1, whereas those to the right have not. Scale bar = 100 μm. (**B**) Effect of different ACE concentrations on the PB1 extrusion rate of porcine oocytes. n = 90. (**C**) Cumulus expansion of COCs from the control group and ACE-treated groups. Scale bar = 500 μm. (**D**) Effects of different ACE concentrations on the cumulus expansion index of COCs. n = 90. (**E**) Normal spindle morphology of oocytes at various meiotic stages. Scale bar = 10 μm. Green: α-tubulin, blue: chromosome. Stages: GV (germinal vesicle), GVBD (germinal vesicle breakdown), MI (metaphase I), ATI (anaphase-telophase I), and MII (metaphase II). (**F**) Proportion of oocytes arrested at different meiotic stages following treatment with different ACE concentrations. n = 90. (**G**) Representative images of spindle morphology and chromosome alignment in oocytes treated with different ACE concentrations. Scale bar = 10 μm. (**H**) Percentage of oocytes with aberrant spindles in groups treated with different ACE concentrations. n = 90. The letter “n” indicates the total number of oocytes in each group of three independent replicates. ^a,b^ Values with different superscripts indicate statistical significance (*p* < 0.05).

As shown in Figure 1A–D, the first polar body (PB1) extrusion and cumulus expansion were markedly inhibited as the ACE concentration increased. Specifically, when the ACE concentration reached 20 and 40 μM, both the PB1 extrusion rate and the cumulus expansion index significantly decreased (*p* < 0.05). Cell cycle analysis further showed that 20 and 40 μM ACE significantly increased the proportion of oocytes arrested at the germinal vesicle breakdown (GVBD) or anaphase-telophase I (ATI) stage (Figure 1E,F). Furthermore, as shown in Figure 1G, oocytes in the control group exhibited a typical barrel-shaped spindle and a polar body, while those in the ACE-treated group displayed aberrant α-tubulin and misaligned chromosomes. The quantitative analysis indicated that the percentage of oocytes with aberrant spindle assembly was significantly increased when the ACE concentration reached 20 μM (Figure 1H, *p* < 0.05).

### 3.2. ACE Exposure Leads to Oxidative Stress and Mitochondrial Impairment in Porcine Oocytes

**Figure 2:**
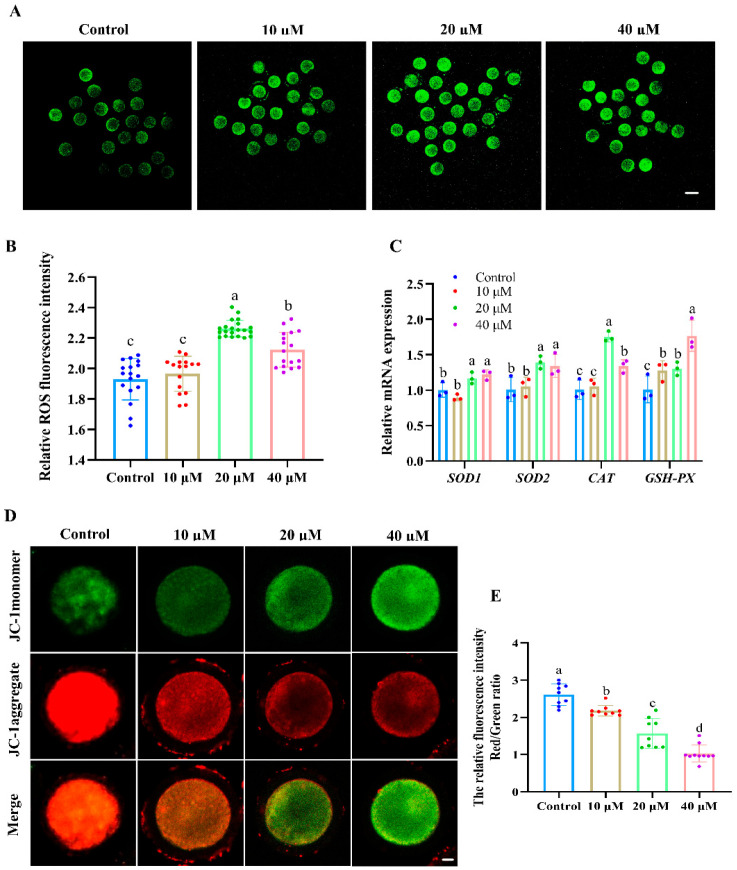
(**A**) Representative images of the ROS fluorescence intensity of oocytes in groups treated with different concentrations of ACE. Scale bar = 100 μm. (**B**) Relative ROS fluorescence intensity in oocytes treated with different concentrations of ACE. n = 60. (**C**) Relative mRNA expression levels in oocytes treated with different concentrations of ACE. n = 300. (**D**) Representative images of JC-1 staining in oocytes from the control and ACE treatment groups. Scale bar = 10 μm. (**E**) Relative fluorescence intensity of red/green signals in oocytes treated with different concentrations of ACE. n = 30. ^a–d^ Values with different superscripts indicate statistical significance (*p* < 0.05).

As shown in Figure 2A,B, the fluorescence intensity of ROS in oocytes was significantly increased in the 20 and 40 μM ACE-treated groups compared with the control group (*p* < 0.05). Moreover, the relative mRNA levels of antioxidant enzymes, including *SOD1*, *SOD2*, *CAT*, and *GSH-px*, were upregulated in the oocytes after 20 and 40 μM ACE exposure (Figure 2C, *p* < 0.05). Furthermore, the results presented in Figure 2D,E show that the MMP levels, as indicated by the red/green fluorescence intensity ratio, decreased in a dose-dependent manner in the oocytes following ACE exposure (*p* < 0.05). These results suggest that ACE exposure induces oxidative stress and mitochondrial damage in oocytes.

### 3.3. ACE Exposure Led to Lipid Peroxidation and Iron Overload in Porcine Oocytes

**Figure 3:**
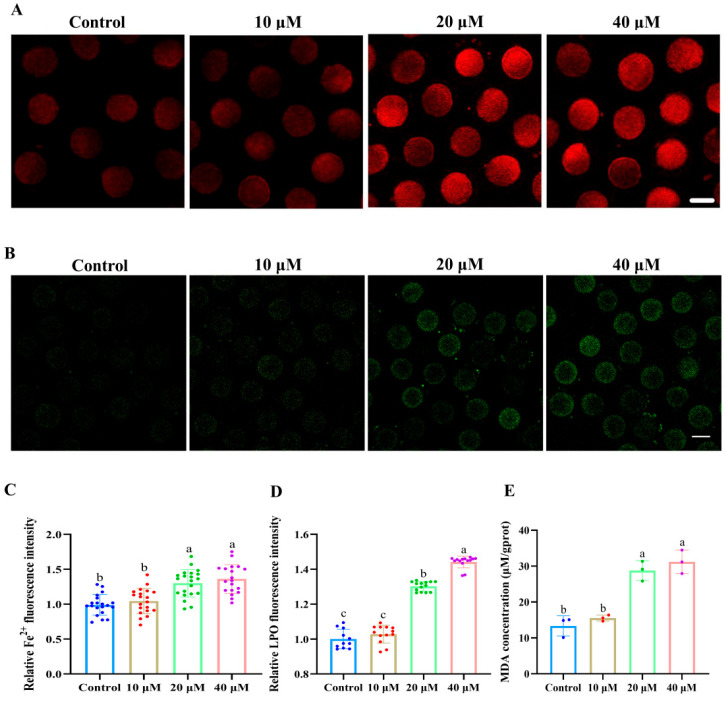
(**A**) Representative images of Fe^2+^ fluorescence intensity in oocytes treated with different concentrations of ACE. Scale bar = 100 μm. (**B**) Representative images of lipid peroxidation (LPO) fluorescence intensity in oocytes treated with different concentrations of ACE. Scale bar = 100 μm. (**C**) Relative Fe^2+^ fluorescence intensity in oocytes treated with different concentrations of ACE. n = 60. (**D**) Relative LPO fluorescence intensity in oocytes treated with different concentrations of ACE. n = 30. (**E**) Malondialdehyde (MDA) concentration of oocytes treated with different concentrations of ACE. n = 1050. ^a–c^ Values with different superscripts indicate statistical significance (*p* < 0.05).

Figure 3A,C show that the fluorescence intensity of Fe^2+^ in 20 and 40 μM ACE-treated oocytes was significantly higher than that in the control group (*p* < 0.05). Meanwhile, the accumulation of LPO and MDA was also increased in 20 and 40 μM ACE-treated oocytes (Figure 3B,D,E, *p* < 0.05). These results suggest that ACE exposure leads to excessive iron accumulation and enhanced lipid peroxidation in porcine oocytes. According to the results above, a concentration of 20 μM ACE was selected for subsequent experiments.

### 3.4. Effects of Fer-1 on Lipid Peroxidation and Iron Overload in ACE-Exposed Oocytes

**Figure 4:**
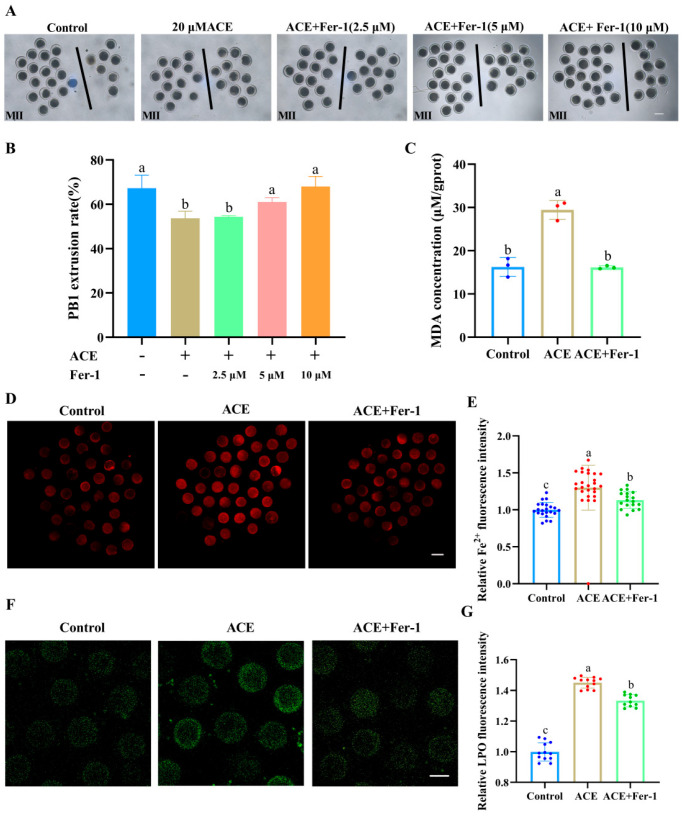
(**A**) The first polar body (PB1) extrusion of oocytes from control, ACE-treated (20 μM), and Fer-1 co-treated (2.5, 5, and 10 μM) groups. Scale bar = 100 μm. (**B**) Effects of Fer-1 co-treatment (2.5, 5, and 10 μM) on PB1 extrusion rate in ACE-exposed (20 μM) oocytes. n = 75. (**C**) Malondialdehyde (MDA) concentration in oocytes from control, 20 μM ACE-treated, and ACE + 5 μM Fer-1 co-treated groups. n = 1050. (**D**) Representative images of Fe^2+^ fluorescence intensity in oocytes from control, 20 μM ACE-treated, and ACE + 5 μM Fer-1 co-treated groups. Scale Bar = 100 μm. (**E**) Relative Fe^2+^ fluorescence intensity in oocytes from control, 20 μM ACE-treated, and ACE + 5 μM Fer-1 co-treated groups. n = 60. (**F**) Representative images of LPO fluorescence intensity in oocytes from control, 20 μM ACE-treated, and ACE + 5 μM Fer-1 co-treated groups. Scale Bar = 100 μm. (**G**) Relative LPO fluorescence intensity in oocytes from control, 20 μM ACE-treated, and ACE + 5 μM Fer-1 co-treated groups. n = 30. ^a–c^ Values with different superscripts indicate statistical significance (*p* < 0.05).

To explore whether the toxic effects of ACE on porcine oocytes were mediated by ferroptosis, Fer-1, a specific small-molecule inhibitor of ferroptosis, was used to cotreat oocytes with ACE. As shown in Figure 4A,B, Fer-1 at concentrations of 5 μM and 10 μM co-treatment significantly increased the PB1 extrusion rate in ACE-exposed oocytes, showing no statistical differences compared to the control group (*p* > 0.05). Based on this result, a concentration of 5 μM of Fer-1 was used for subsequent experiments.

Moreover, the levels of Fe^2+^ as well as the accumulation of LPO and MDA were significantly decreased in the Fer-1 co-treatment group (Figure 4C–G, *p* < 0.05). Meanwhile, the ACE-induced alterations in the expression of ferroptosis-related genes and proteins, including ACSL4, TfR1, GPX4, and SLC7A11, were reversed following Fer-1 co-treatment (Figure S1). These results suggest that ACE exposure induces lipid peroxidation and ferroptosis in porcine oocytes.

### 3.5. Effects of α-TOC on the Meiotic Maturation and Oxidative Stress of ACE Exposed-Oocytes

**Figure 5:**
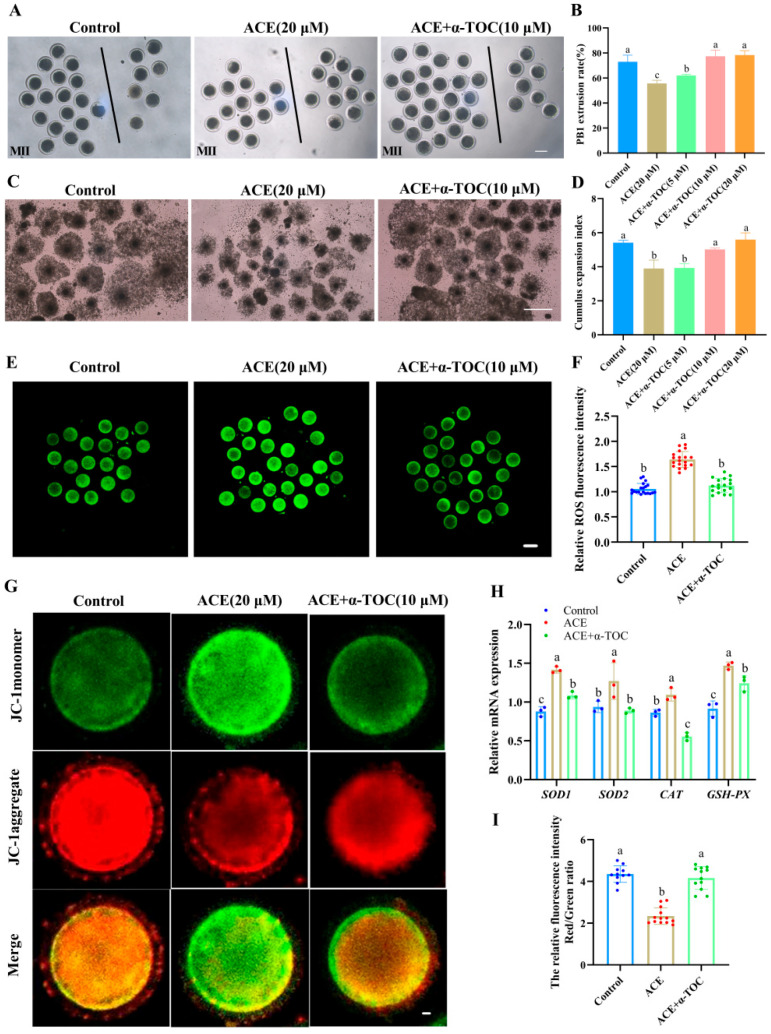
(**A**) Representative images of the first polar body (PB1) extrusion of oocytes from the control group, 20 μM ACE, and ACE + 10 μM α-TOC co-treated groups. Scale bar = 100 μm. (**B**) Effects of different concentrations of α-TOC (5, 10, and 20 μM) on the PB1 extrusion rate of ACE-exposed (20 μM) oocytes. n = 75. (**C**) Cumulus expansion of cumulus oocyte complexes (COCs) from the control, 20 μM ACE, and ACE + 10 μM α-TOC co-treated groups. Scale bar = 500 μm. (**D**) Effects of different concentrations of α-TOC (5, 10, and 20 μM) on the cumulus expansion index of ACE-exposed (20 μM) COCs. (**E**) Representative images of ROS staining in oocytes from control, 20 μM ACE-treated, and ACE + 10 μM α-TOC co-treated groups. Scale bar = 100 μm. (**F**) Relative fluorescence intensities of ROS signals in oocytes from control, 20 μM ACE-treated, and ACE + 10 μM α-TOC co-treated groups. n = 60. (**G**) Representative images of JC-1 staining in oocytes from control, 20 μM ACE-treated, and ACE + 10 μM α-TOC co-treated groups. Scale bar = 10 μm. (**H**) Effect of α-TOC on the mRNA expression levels of antioxidant-related genes in ACE-exposed oocytes. n = 300. (**I**) Relative average fluorescence intensities of JC-1 red/green signals in oocytes from control, 20 μM ACE-treated, and ACE + 10 μM α-TOC groups. n = 30. ^a–c^ Values with different superscripts indicate statistical significance (*p* < 0.05).

To explore whether α-TOC could alleviate the adverse effects of ACE on porcine oocytes, α-TOC was supplemented into the IVM culture medium containing 20 μM ACE. As shown in Figure 5A,B, co-treatment with either 10 or 20 μM α-TOC significantly restored the PB1 extrusion rate of ACE-exposed oocytes to levels comparable with the control group (*p* < 0.05). Furthermore, α-TOC co-treatment (10 and 20 μM) effectively reversed the ACE-induced inhibition of cumulus expansion in oocytes (Figure 5C,D). According to these results, 10 μM α-TOC was selected for the subsequent experiments.

The ROS analysis results showed that the fluorescence intensity of ROS was markedly decreased in the α-TOC co-treatment group (Figure 5E,F, *p* < 0.05). Meanwhile, the up-regulated mRNA levels of *CAT*, *GPX2*, *SOD1*, and *SOD2* in the ACE-exposed oocytes were effectively alleviated by α-TOC co-treatment. (Figure 5H, *p* < 0.05). Furthermore, the MMP levels of ACE-exposed oocytes were also increased after α-TOC co-treatment (Figure 5G,I, *p* < 0.05).

### 3.6. Effect of α-TOC on Ferroptosis in ACE Exposed-Oocytes

**Figure 6:**
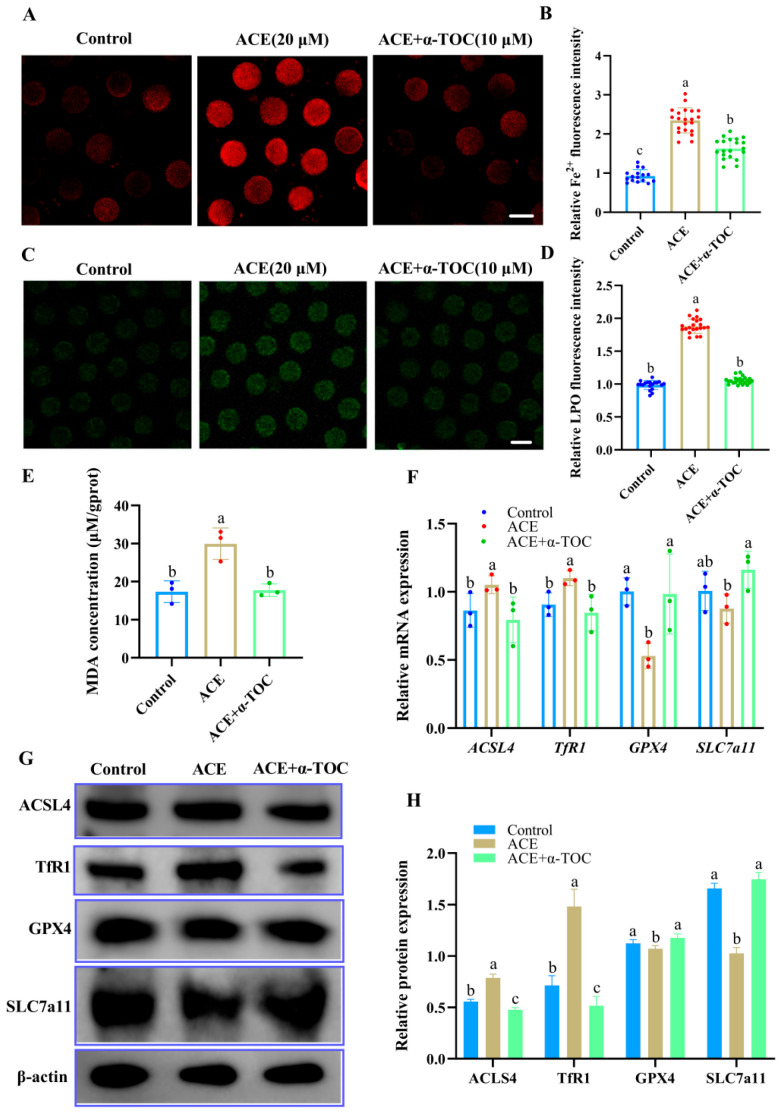
(**A**) Representative images of Fe^2+^ fluorescence intensity in oocytes from control, 20 μM ACE-treated, and ACE + 10 μM α-TOC co-treated groups. Scale Bar = 100 μm. (**B**) Relative Fe^2+^ fluorescence intensity in oocytes from control, 20 μM ACE-treated, and ACE + 10 μM α-TOC co-treated groups. n = 60. (**C**) Representative images of LPO fluorescence intensity in oocytes from control, 20 μM ACE-treated, and ACE + 10 μM α-TOC co-treated groups. Scale Bar = 100 μm. (**D**) Relative LPO fluorescence intensity in oocytes from control, 20 μM ACE-treated, and ACE + 10 μM α-TOC co-treated groups. n = 60. (**E**) MDA concentration in oocytes from control, 20 μM ACE-treated, and ACE + 10 μM α-TOC co-treated groups. n = 1050. (**F**) Relative mRNA expression levels of ferroptosis-related genes in oocytes from the control, 20 μM ACE-treated, ACE + 10 μM α-TOC co-treated groups. n = 300. (**G**,**H**) Expression of ferroptosis-related proteins in oocytes from the control, 20 μM ACE-treated, and ACE + 10 μM α-TOC co-treated groups. n = 450. ^a–c^ Values with different superscripts indicate statistical significance (*p* < 0.05).

To further determine whether α-TOC could mitigate ferroptosis in ACE-exposed oocytes, the levels of Fe^2+^, LPO, and MDA in the oocytes were evaluated, and the expression of ferroptosis-associated genes and proteins was also analyzed after α-TOC co-treatment. As shown in Figure 6A–E, α-TOC co-treatment significantly decreased the accumulation of Fe^2+^, LPO, and MDA in the ACE-exposed oocytes (*p* < 0.05). Furthermore, the qRT-PCR and western blot results revealed that α-TOC co-treatment effectively reversed the alterations in ferroptosis-associated gene and protein expression induced by ACE exposure (Figure 6F–H, *p* < 0.05).

## 4. Discussion

Acetamiprid, a widely used insecticide in agricultural and domestic settings, has garnered considerable attention due to its reproductive toxicity in mammals. The results of this study indicate that ACE exposure could induce ferroptosis in porcine oocytes by promoting oxidative stress and lipid peroxidation, eventually resulting in failure of oocyte maturation. Importantly, α-TOC co-treatment exerts a positive protective effect against ACE-induced meiotic defects, suggesting its potential as a protective agent. This study provides the first direct evaluation of ACE-induced oocyte toxicity using a porcine in vitro maturation model. Compared with mouse oocytes, porcine oocytes share greater structural and biochemical similarity with human oocytes, particularly in terms of chromosomal organization and lipid composition, thereby offering a more translationally relevant system [32]. Moreover, our findings present the first evidence that ferroptosis contributes to ACE-induced meiotic failure in mammalian oocytes. We further establish that α-TOC effectively alleviates this damage by reducing oxidative stress and suppressing ferroptosis, thus preserving oocyte quality. These findings offer direct experimental support for the potential of dietary antioxidant interventions in mitigating pesticide-induced reproductive toxicity.

Previous studies have shown that prolonged environmental or accidental exposure to ACE can alter hematological, biochemical, and structural parameters, leading to neurological, hepatorenal, immunological, genotoxic, and reproductive impairments [33]. These adverse effects are frequently associated with long-term cumulative toxicity. Considering that porcine oocyte IVM lasts approximately 44 h, the present study employed acute ACE exposure throughout this meiotic window to simulate the potential risk of chronic accumulation. The chosen concentrations were designed to elicit measurable biological responses and establish a dose-dependent relationship. This strategy enabled the assessment of ACE’s detrimental effects on female germ cells, the identification of its potential cellular targets, and the provision of molecular-level evidence supporting the environmental risk evaluation of ACE exposure.

PB1 extrusion and complete cumulus expansion are established morphological indicators of oocyte meiotic maturation [34]. Our results demonstrate that ACE exposure significantly impaired porcine oocyte maturation, as evidenced by reduced PB1 extrusion rates and diminished cumulus expansion indices. These findings corroborate previous reports of ACE’s detrimental effects on oocyte maturation, including impaired nuclear maturation in porcine oocytes [35] and disrupted meiotic progression in murine models [13], confirming a conserved inhibitory effect across mammalian species. Proper assembly of the spindle is critical for oocyte meiotic maturation [36,37]. To further explore the reasons for the failure of oocyte maturation following ACE exposure, the cell cycle progression and meiotic spindle structure of the oocytes were evaluated. The disrupted cell cycle and aberrant spindles were observed in the ACE-exposed group, suggesting that ACE could interfere with spindle assembly and cell cycle progression of the oocytes during in vitro maturation, thereby contributing to the impaired maturation of oocytes.

ATP generation by mitochondria is crucial for providing the energy required for meiosis in oocytes [38], and mitochondrial dysfunction could cause the failure of oocyte maturation [39]. MMP serves as an important indicator of mitochondrial function [40], with oocytes possessing high developmental potential, typically exhibiting higher MMP levels [41]. Therefore, we further evaluated the MMP in the oocytes to confirm whether ACE exposure would impair mitochondrial function. The results showed that ACE treatment increased MMP depolarization, suggesting the occurrence of mitochondrial dysfunction. Consistent with our findings, Annabi et al. demonstrated that ACE induces a loss of MMP in rat pheochromocytoma (PC12) cells [42]. Similarly, Kong et al. found that ACE exposure caused mitochondrial membrane damage in rat Leydig cells, which disrupted testosterone biosynthesis and led to reproductive damage [10].

Mitochondria naturally generate ROS as metabolic byproducts, with homeostasis maintained through transient openings of the mitochondrial permeability transition pore (mPTP). Under oxidative stress, prolonged mPTP opening leads to excessive ROS production, establishing a vicious cycle of ROS bursts and mitochondrial damage [43]. Both oxidative stress and mitochondrial deficits have been shown to cause failure of oocyte maturation [25,39]. In this study, the observed decrease in ROS levels at 40 µM ACE may reflect severe cellular injury. While 20 µM ACE induced detectable oxidative stress, the higher concentration (40 µM) likely caused extensive mitochondrial and metabolic damage, thereby diminishing overall ROS-generating capacity and leading to this paradoxical reduction.

The cellular antioxidant defense system, comprising key enzymes including superoxide dismutase (SOD), catalase (CAT), and glutathione peroxidase (GPX), plays a critical role in maintaining redox homeostasis and protecting against oxidative damage [44,45,46]. Our findings demonstrate that ACE exposure induces oxidative stress in porcine oocytes, as evidenced by elevated ROS levels and upregulated mRNA expression of antioxidant enzymes (*SOD*, *CAT*, *GPX*, and *GSH-Px*). This aligns with previous reports identifying oxidative stress as a primary mechanism of ACE toxicity [2]. Consistent with the findings of this study, Gasmi et al. showed that ACE treatment led to mitochondrial damage, increased ROS levels, and increased antioxidant enzyme SOD activity in rat brain tissue [47]. The observed upregulation of antioxidant enzymes in oocytes following ACE treatment likely represents a compensatory cellular stress response to counteract excessive superoxide production and maintain redox homeostasis.

Excessive ROS can deplete intracellular oxygen and trigger peroxidation of membrane polyunsaturated fatty acids (PUFAs), leading to lipid peroxidation [48]. The accumulation of lipid peroxides to lethal levels could induce ferroptosis [49,50,51], an iron-dependent cell death process known to impair porcine oocyte developmental competence and cause in vitro maturation failure [52]. Based on these mechanisms, we hypothesized that ACE-induced oxidative stress would trigger lipid peroxidation and subsequent ferroptosis in porcine oocytes. Consistent with this, ACE-exposed oocytes exhibited significant accumulation of Fe^2+^, LPO, and MDA, confirming the induction of ferroptosis. Co-treatment with the specific ferroptosis inhibitor Fer-1, at concentrations informed by previous studies [53], significantly attenuated these effects and improved oocyte maturation rates. Collectively, these results suggest that ACE-induced impairment of oocyte maturation is mediated, at least in part, by ferroptosis. These results align with previous reports documenting ACE-induced lipid peroxidation in cord blood erythrocytes and elevated MDA levels in rat PC12 cells [42,54] collectively supporting the pathway whereby ACE-induced oxidative stress initiates ferroptosis. Nevertheless, given that ACE-induced ROS and mitochondrial damage may also activate additional cell death pathways, further investigation is warranted to fully elucidate the underlying mechanisms.

To further elucidate the mechanism of ACE-induced oocyte toxicity, the expression levels of ferroptosis-related genes were examined in this study. ACE exposure decreased the expression of SLC7A11 and GPX4 while upregulating ACSL4 and TfR1 in porcine oocytes. TfR1 mediates the intracellular transport of Fe^2+^, which is essential for the Fenton reaction, thereby promoting ferroptosis [55]. ACSL4, a key synthase for PUFAs, contributes to ferroptosis by driving excessive PUFA production [49]. Conversely, SLC7A11 and GPX4, critical components of the antioxidant defense system, protect cells from ferroptosis by scavenging lipid peroxides [56]. Collectively, these findings suggest that ACE induces ferroptosis by upregulating ACSL4 and TfR1 while suppressing SLC7A11 and GPX4 expression.

α-TOC, a potent peroxyl radical scavenger, exerts its antioxidant effects by neutralizing peroxyl radicals prior to their interaction with PUFAs [57]. Due to its ability to mitigate oxidative stress and cellular damage, α-TOC has been widely employed in biomedical research. It has been demonstrated that α-TOC inhibits phoxim-induced mitochondrial apoptosis in renal PK12 cells of piglets, reduces intracellular ROS levels, and modulates the expression of antioxidant enzymes (SOD and CAT) [58]. It also promotes in vitro oocyte maturation under heat stress in dairy cows [31] and ameliorates ACE-induced oxidative stress and mitochondrial damage in Leydig cells, restoring testosterone levels and semen quality in rats [10]. Consistently, our study reveals that α-TOC restores PB1 expulsion and cumulus expansion, thereby improving porcine oocyte maturation by protecting oocytes from oxidative stress and mitochondrial dysfunction induced by ACE exposure. These results indicate that α-TOC is a potential candidate for improving oocyte maturation in ACE-exposed oocytes.

α-TOC, which plays a crucial role in protecting PUFAs against oxidative damage [59], has been found to attenuate radiation-induced MDA accumulation, lipid peroxidation, and intracellular iron levels, thereby mitigating radiation injury by inhibiting ferroptosis in hippocampal neurons in mice [60]. Our findings are consistent with these observations, showing that α-TOC treatment effectively alleviates lipid peroxidation and ferroptosis in porcine oocytes. Beyond its well-established radical-scavenging capacity, α-TOC has been shown to modulate the expression of ferroptosis-related genes [61]. Zhang et al. demonstrated that α-TOC suppresses neuronal ferroptosis by increasing GPX4 expression [62]. Meanwhile, Hu et al. reported that α-TOC protects hematopoietic stem and progenitor cells from lipid peroxidation and ferroptosis in mice with GPX4 deficiency [63]. Our data complement these reports by demonstrating that α-TOC co-treatment upregulated GPX4 and SLC7A11 while downregulating ACSL4 and TfR1 expression. These effects were consistent with the reversal observed with the ferroptosis inhibitor Fer-1, supporting the characterization of α-TOC as an effective, albeit non-specific, inhibitor of ferroptosis [51,64]. Notably, dietary supplementation of 100–200 IU kg^−1^ α-TOC has been shown to elevate serum α-TOC to concentrations between 15 and 25 µM, a range associated with improved litter size in sows [21]. Our in vitro data demonstrate that α-TOC confers protection against ACE-induced damage in porcine oocytes at a physiologically relevant concentration (10 µM), providing mechanistic support for this observation. Collectively, these findings suggest that maintaining maternal dietary vitamin E levels may represent a practical nutritional strategy to mitigate oocyte quality deterioration caused by environmental ACE residues.

## 5. Conclusions

In conclusion, our findings demonstrate that ACE exposure compromises porcine oocyte quality by disrupting spindle assembly and perturbing cell cycle progression during in vitro maturation, ultimately leading to meiotic maturation failure. Importantly, α-TOC cotreatment effectively rescues ACE-induced oocyte defects by mitigating oxidative stress, attenuating lipid peroxidation, and suppressing ferroptosis. These results provide novel insights into the protective role of α-TOC against ACE-mediated oocyte damage and suggest its potential as a therapeutic intervention for ACE-associated reproductive toxicity.

## Data Availability

The original contributions presented in this study are included in the article and Supplementary Materials. Further inquiries can be directed to the corresponding authors.

## Supplementary Materials

The following supporting information can be downloaded at https://www.mdpi.com/article/10.3390/antiox14111304/s1. Figure S1. (**A**,**B**) Expression of ferroptosis-related proteins in oocytes from the control, 20 μM ACE-treated, and ACE + 5 μM Fer-1 co-treated groups. n = 450. (**C**) Relative mRNA expression levels of ferroptosis-related genes in oocytes from the control, 20 μM ACE-treated, and ACE + 5 μM Fer-1 co-treated groups. n = 300. ^a-c^ Values with different superscripts indicate statistical significance (*p* < 0.05); Table S1: Primer sequences used for quantitative real-time PCR.
