# Supplementary material for: Alpha-Tocopherol Protects Porcine Oocytes from Acetamiprid-Induced Meiotic Defects by Alleviating Oxidative Stress-Mediated Ferroptosis
Source: Antioxidants (Basel). 2025 Oct 30;14(11):1304. doi: 10.3390/antiox14111304 (PMC12649341; doi:10.3390/antiox14111304)
Supplement: Supplementary file 1 [file antioxidants-14-01304-s001.zip › Table S1.pdf]

Table S1

Primer sequences used for quantitative real-time PCR

Gene	Primer Sequence (5′-3′)	Fragment size (bp)	Access number
β-actin	F-5'-TCAACACCCCAGCCATGTAC-3'<br>R-5'-CTCCGGAGTCCATCACGATG-3'	147	XM_021086047.1
CAT	F-5'-AACTGTCCCTTCCGTGCTA-3'<br>R-5'-CCTGGGTGACATTATCTTCG-3'	195	NM_214301.2
GSH-PX	F-5'-CAAGTCCTTCTACGACCTCA-3'<br>R-5'-GAAGCCAAGAACCACCAG-3'	210	NM_214201.1
SOD1	F-5'-ACCTGGGCAATGTGACTG-3'<br>R-5'-TCCAGCATTTCCCGTCT-3'	197	NM_001190422.1
SOD2	F-5'-GGACAAATCTGAGCCCTAACG-3'<br>R-5'-CCTTGTTGAAACCGAGCC-3'	184	NM_214127.2
ACSL4	F-5'-GGAAGTCCATATCGCTCTGTCACAC-3'<br>R-5'-CTCCCTGGTCCCAAGGCTGTC-3'	135	XM_005673819.3
Tfr1	F-5'-TGGCTCGGCAGGTAGATGGTG-3'<br>R-5'-TGTGGTTACTCCTTGTGTTGCTGTC-3'	96	NM_214001.1
SLC7a11	F-5'-TCTTTGTTGCCCTCTCCTGCTTTG-3'<br>R-5'-GTGTGTTTGCGGATGTGAATCATGG-3'	130	XM_021101587.1
GPX4	F-5'-GCCTGTTCCGCCTGCTGAAG-3'<br>R-5'-CATGTGCCCGTCGATGTCCTTG-3'	146	NM_214407.1
