# Supplementary figures and images for: Alpha-Tocopherol Protects Porcine Oocytes from Acetamiprid-Induced Meiotic Defects by Alleviating Oxidative Stress-Mediated Ferroptosis
Source: Antioxidants (Basel). 2025 Oct 30;14(11):1304. doi: 10.3390/antiox14111304 (PMC12649341; doi:10.3390/antiox14111304)

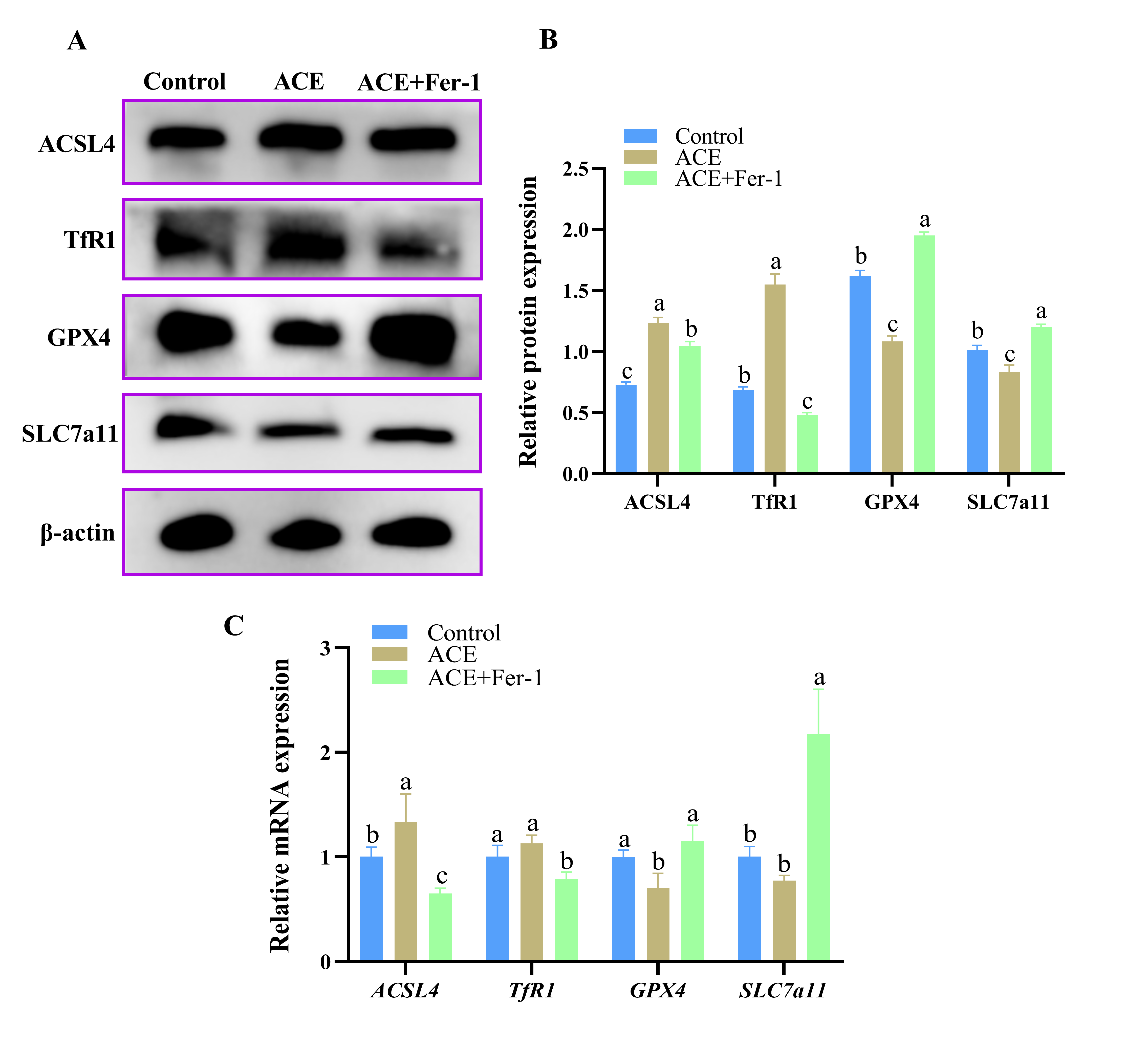

Supplement: Supplementary file 1 [file antioxidants-14-01304-s001.zip › Figure S1.tif]
